# Malignant transformation rate of oral precancerous disorders to oral cancer: systematic review and meta-analysis of the current evidence

**DOI:** 10.3389/froh.2025.1673474

**Published:** 2025-10-02

**Authors:** Maryam Khodadadi, Mohammadmehdi Khodadadi

**Affiliations:** 1 School of Stomatology, Xi'an Jiaotong University, Xi'an, Shaanxi, China; 2 Medical Campus, Zhengzhou University, Zhengzhou, Henan, China

**Keywords:** OPMDs, oral cancer, malignant, systematic review, meta-analysis

## Abstract

**Background:**

Oral potentially malignant disorders (OPMDs) are precancerous conditions that may eventually turn into oral cancer (OC). The aim of this present study was to reassess the data associated with OPMDs and their malignant transformation rate into oral cancer.

**Methods:**

A comprehensive search was performed in PubMed, Scopus, Embase, and Web of Science (WoS) to locate studies on OPMDs risk of OC up to July 10, 2025. A summary odds ratio (SOR) was used for the analysis of data. To explore the heterogeneity, subgroup analyses and meta-regression analyses were used. The certainty of evidence was rated using the GRADE protocol.

**Results:**

Twenty-nine studies met the inclusion criteria. The meta-analysis showed an elevated risk of OC by overall OPMDs (SOR = 2.5 (95% CI 2.43–2.58). The highest risk of OC in OPMDs were leukoplakia (SOR = 3.35 (95% CI 3.21–3.50), erythroplakia (SOR = 3.3 (95% CI 3.21–3.50), oral epithelial dysplasia (SOR = 1.41 (95% CI 1.20–1.73), and oral submucosa fibrosis (SOR = 2.9 (95% CI 2.70–3.12). Subgroup and meta-regression analyses demonstrated a significant positive association between the development risk of OC with age and duration of follow up.

**Conclusions:**

This study suggests a significant association between OPMDs and OC. Age and duration of follow up may modify this association. This study provides insight into the malignant transformation rate to oral cancer to aid researchers, dentists, and clinicians in efficiently controlling this disease in the general population.

## Introduction

The upkeep of oral health is a primary obstacle in the prevention of oral cancer (OC). OC is a severe and incapacitating illness of the oral cavity that continues to be the most prevalent site for cancer in many countries (1). The majority of these OCs are squamous cell carcinomas, while other forms of oral malignancies indicate a significantly smaller percentage (2, 3). OC is one of the most common malignant cancers in the head and neck region. Based on the Global Cancer Statistics Database (GLOBOCAN) from the World Health Organization's International Agency for Research on Cancer (WHO/IARC) for the year 2022, OC is positioned 15th in terms of global average mortality, resulting in 188,438 deaths, namely 130,808 deaths among males and 57,630 among females (World Health Organization 2024). The five-year survival rate for individuals diagnosed with OC is roughly between 40% and 50%, and numerous survivors may encounter a considerable decline in their quality of life (4). In addition, in the Global Cancer Observatory (GCO) showed that, among all continents, Asia exhibits the highest prevalence of OC (which includes lip and tongue cancers), accounting for 65.8% of all cases. This is followed by Europe at 17.3% and North America at 7.3% (5). Early detection and management of oral premalignant lesions are crucial for preventing OC and improving patient survival. Continuous exposure to a variety of risk factors, including tobacco, alcohol, betel quid (BQ), and human papillomavirus (HPV), can result in the emergence of OPMDs. These disorders are characterized by oral mucosal lesions that carry a heightened risk of progressing to oral squamous cell carcinomas (OSCC) (6). Many studies have shown that patients with oral premalignant lesions present a high risk of developing oral malignancies such as OSCC (7, 8).

Oral Potentially Malignant Disorders (OPMDs) refer to conditions affecting the oral mucosa that present an increased risk of developing into OC. The common OPMDs include oral leukoplakia (OL), oral erythroplakia (OE), oral lichen planus (OLL), oral epithelial dysplasia (OED), and oral submucous fibrosis (OSF) (9, 10). The malignant transformation rates (MTR) of OPMDs to OC are usually related to risk factors such as genetics, geographic variation, and lifestyle factors (7, 11, 12). Multiple studies have indicated the malignant transformation tendencies of OPMD subtypes according to the MTR (13–15). The categorization of OPMD subtypes through MTR could aid in the formulation of subsequent strategies and the improvement of treatment methods, thereby reducing the risk of malignant transformation. MTR has been documented as 3% for homogeneous lesions and 14.5% for non-homogeneous lesions (16). Furthermore, other studies have reported the degree of MTR as 0%–3.0% for mild (low risk of dysplasia lesion), 4%–15% for moderate (moderate risk of dysplasia lesion), and >15% for severe dysplasia lesions (high risk of dysplasia lesion) (17). According to the MTR, proliferative verrucous leukoplakia (PVL) with 49.5% and OE with 14%–85% have the highest incidence rates. The MTR for OLP, OLL, and OSF is 1.14%, 1.71%, and 4.2%, respectively (16). A major OPMD is oral leukoplakia (OL), a form of which is proliferative verrucous leukoplakia (PVL). Hansen et al. first defined proliferative verrucous leukoplakia (PVL) in 1985. PVL is related to a strong affinity to recur after treatment and an increased risk of OSCC (18, 19). PVL is defined by a gradual advancement and a slowly evolving clinical trajectory, accompanied by both clinical and histopathological alterations. It represents a severe form of oral leukoplakia that is associated with significant morbidity and a pronounced tendency towards malignant transformation (20). Multiple systematic reviews and meta-analyses have shown the high transformation rate of oral leukoplakia to oral cancer (21–24). Another important OPMD is oral erythroplakia (OE). The clinical presentation of OE is characterized by a distinctly defined, solitary red lesion on the oral mucosa, which may be situated at a lower elevation compared to the adjacent mucosal tissue (25, 26). The developed OE lesion has a bright (fiery) red color with a matte smooth surface and is velvety or granular appearance. The common locations for OE in the mouth are the soft palate, floor of the mouth, and the buccal mucosa (27, 28). Oral epithelia dysplasia (OED), oral submucous fibrosis (OSF), and oral lichen planus (OLP) are other examples of OPMDs (29). The role of OPMDs as risk factors initiating the development of oral malignant cancer has been previously investigated in individuals with OPMDs (15, 30, 31). Intriguingly, studies in individuals with OPMDs have shown conflicting results. This systematic review and meta-analysis aims to synthesize current evidence to quantify the malignant transformation risks of various OPMDs and explore the incidence rate of OC.

## Methods

The protocol phases of this systematic review and meta-analysis were executed in alignment with the Preferred Reporting Items for Systematic Reviews and Meta-Analyses (PRISMA) in a sequential manner (32).

### Process of selecting studies

The articles that were published underwent a two-step screening procedure for their inclusion in the systematic review and meta-analysis, with any discrepancies resolved through discussion at each phase with a third investigator to achieve a consensus. In addition, we considered studies that presented the OPMDs along with the development risk of OC. Studies were evaluated according to specified inclusion and exclusion criteria.

### Inclusion and exclusion criteria

Studies were considered eligible for inclusion if the OPMDs and the risk of OC were reported in groups. The articles were excluded if they (1) were published as brief reports, opinions, perspectives, book chapters, review articles, or editorials; (2) included other subjects such as animals or *in vitro* subjects; (3) did not provide full access to the complete text; (4) were articles with a lack of specific or thorough information regarding OPMDs and the risk of OC; or (5) discussed OC without OMPDs. To develop a thorough and dependable search approach, the sources of the relevant studies were carefully reviewed and assessed. The EndNote X9.1 application was used to remove duplicates. To find appropriate studies, two authors thoroughly evaluated the relevant articles. This evaluation began with an initial examination of the title and summary, followed by a comprehensive analysis of the full-text articles. Disagreements concerning the idea of inclusion were successfully resolved by engaging in discussions and decision-making, which included the involvement of two additional investigators.

### Systematic search of electronic databases

Published articles with different languages were considered. Two biostatisticians, Maryam Khodadadi (M. Kh) and Mohammadmehdi Khodadadi (MM. Kh), with expertise in systematic reviews and meta-analyses, performed the systematic search strategy. A systematic search of previously published studies was performed via PubMed, Scopus, Embase, and Web of Science (WoS) electronic databases up to July 10, 2025. Syntaxes differed according to the database. Keywords for the PubMed search included (“OPMD OR oral potentially malignant cancer OR oral disorder* OR Leukoplakia OR Erythroplakia OR oral submucous fibrosis OR oral lichenoid lesion OR oral epithelial dysplasia OR lichen planus”) AND [“oral cancer OR mouth cancer OR mouth neoplasms OR oral squamous cell carcinoma (OSCC)”], in WoS: [Topic searching (TS) = OPMD OR oral disorder* OR oral mucosa disorder*] AND (TS = oral cancer OR mouth neoplasms OR oral squamous cell carcinoma (OSCC), for Scopus: Title = OPMDs and (Title: oral cancer* OR oral squamous cell carcinoma), and for Embase: Leukoplakia OR Erythroplakia OR oral submucous fibrosis OR oral lichenoid lesion OR oral epithelial dysplasia OR lichen planus AND (oral cancer or oral malignant disorder or mouth malignant, OSCC). Furthermore, a manual search was conducted on grey literature, preprint servers, conference abstracts, and reference lists to uncover potentially relevant studies as well as related reviews and editorials. In addition to the two aforementioned authors, a third independent reviewer assessed the potential eligibility of the articles. Studies with combined data of OPMDs and the risk of OC were utilized solely in the pooled analysis when adequate data for extraction was accessible. The search queries used in each database are available in Box 1 of the Supplementary File.

### Screening of studies and their selection

Two reviewers (M. Kh and MM. Kh) independently assessed the process of study selection by screening all abstracts and titles. Articles were considered in the full text if either reviewer diagnosed the study as potentially eligible or if the abstract and title lacked sufficient information. Studies were eligible for full text screening if they presented oral cancer or malignant transformation rates in patients with OPMDs. OPMDs included Leukoplakia, Erythroplakia, OED, OLL, LP, and OSF. The other investigator independently conducted full text screening to select articles for incorporation based on the criteria listed in the inclusion and exclusion criteria. Disagreements were resolved by consensus or arbitration.

### Data summarization

Two authors independently abstracted the following information from the studies that were incorporated. To achieve this objective, a pre-configured standardized Excel spreadsheet was utilized to gather information. The following data were extracted: the first author's name, publication year, country, No. case, total population, sex (Male/Female), mean age (year), study type, duration of follow up (month), type of lesion, etiological factors, clinicopathological diagnosis, transformation time to malignancy (month), and malignant transformation rate (MTR). With respect to the observational studies, the adjusted odds ratio was calculated according to the number of individuals with OPMD that transformed to OC (exposed) compared to the number of individuals with OPMD without transformation to OC (non- exposed). An impartial reviewer confirmed all data entries and conducted a minimum of two checks to ensure both inclusiveness and accuracy. To access the missing or unclear data, the authors contacted the corresponding author to obtain missing quantitative data.

### Assessment of methodology quality

The quality of the selected articles was evaluated by two reviewers independently. Each reviewer assessed the quality of eligible studies using a consistent version of the Newcastle-Ottawa scale (NOS) tool for observational studies (33). This scale is composed of three domains, namely selection (four points), comparability (two points), and outcome (three points), with a total score of 9 points. Studies scoring 0–5, 5–7, and ≥8 points were detected as having low, moderate, and high risk of bias, respectively. The reviewers addressed any discrepancies in their evaluations through discussion and a third investigator.

### Confidence of evidence

Regarding the main outcome, we assessed the quality of evidence using the GRADE methodology; this analysis accounts for the limitations of the study, the consistency of the observed effects, the degree of imprecision, the potential for indirectness, the presence of publication bias, and the magnitude of the effect, all of which may contribute to minimizing the overall impact.

### Data analysis

Study-data estimates were pooled using a DerSimonian and Laird random-effects meta-analysis model. The strength of correlation between OPMDs and the risk of OC was measured with summary odds ratios with a 95% confidence interval (95% CI). Heterogeneity was evaluated by the χ^2^ test on Cochrane's Q statistic and I^2^ value. The categorization of heterogeneity was based on the Cochrane Handbook, which specifies that I^2^ or Higgins values below 30%, between 30% and 60%, and above 60% are indicative of low, moderate, and high heterogeneity, respectively (34). We estimated the robustness of the results with sensitivity analyses including studies with a low risk of bias to a high risk of bias (35). We performed subgroup analyses according to age [I^2^ (%) 60 and >60 years], duration of OPMD follow up (<60 and ≥60 months), transformation time to oral cancer (<60 and >60 months), MTR (<10 and >10%), study design (cohort, case-control, cross-sectional, or descriptive), and geographical region (Europe, America, or Asia). We identified the publication bias through a visual inspection of funnel plots and the Egger test, with *p* < 0.10 indicating significant publication (36). To take into account the publication bias detected in the global analyses, we conducted adjusted analyses by the Trim-and-fill method. Furthermore, a random-effects meta-regression analysis was conducted utilizing an unrestricted maximum likelihood method to find the effect of covariates such as age, duration of OPMD follow up, transformation time to oral cancer, MTR, study design, and geographical region on the association between OPMDs and the risk of OC. A *P* < 0.05 was considered statistically significant. Comprehensive Meta-Analysis (CMA) V3 software (Biostat, NJ) was used for the overall analysis of extracted data.

## Results

### Process of systematic review

**Figure 1:**
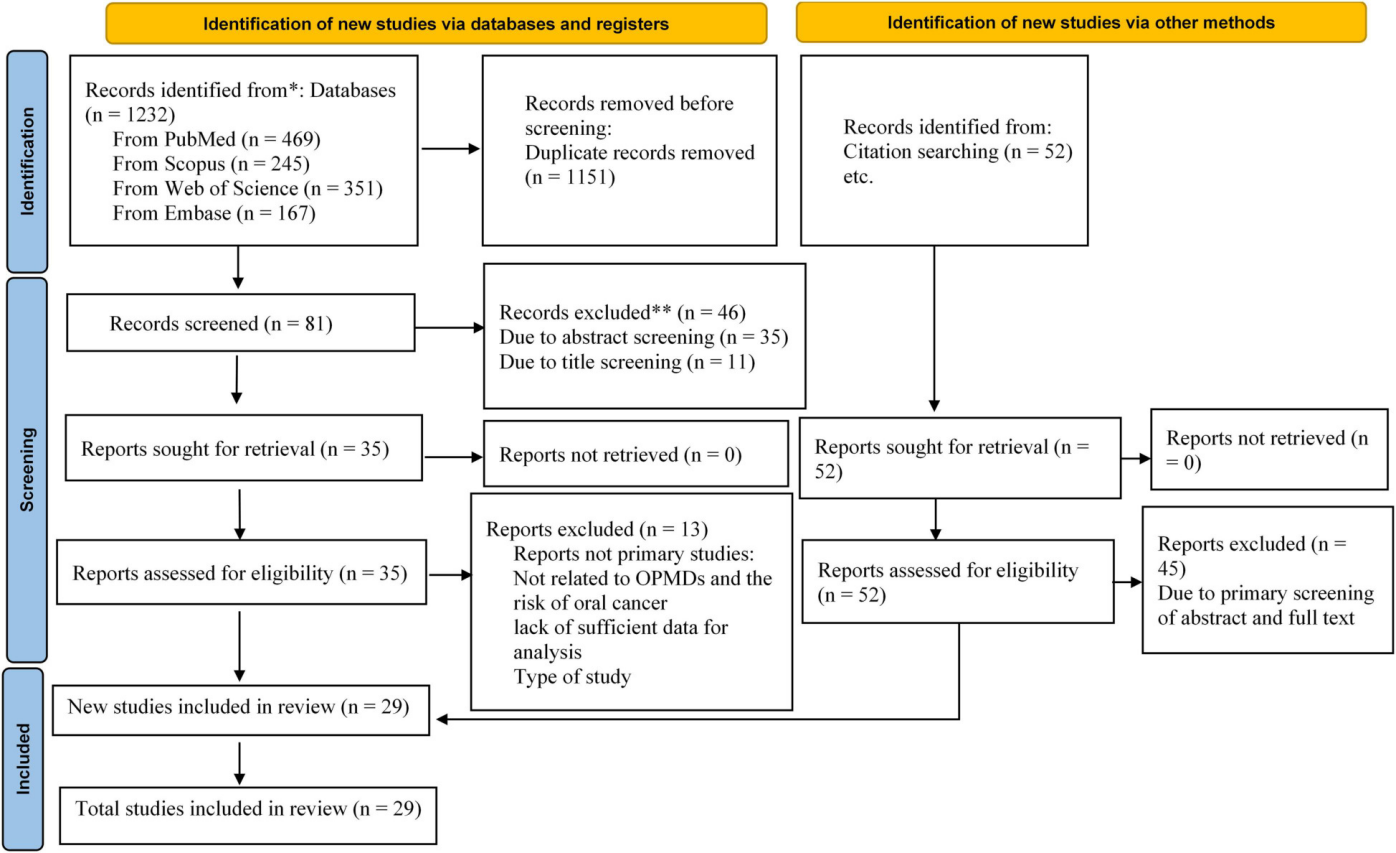
Flowchart of the selection process of studies included in the meta-analysis using PRISMA.

A total of 1,232 potential articles were found from the electronic databases [PubMed (*n* = 469), Scopus (*n* = 245), Web of Science (*n* = 351) and Embase (*n* = 167)]. After the deletion of duplicates, 81 articles remained. Then, after the screening of the titles and abstracts of full-texts, 46 other articles were removed, leaving 35 articles remaining. After searching the reference lists of relevant articles, 52 more articles were recorded. Of those, 45 articles were omitted as they were not original articles, were OPMDs without a report of the risk of oral cancer, studied models other than human such as animals and cell lines, did not present the OPMDs, or were brief reports, editorial letters, or abstracts. The final screening and eligibility was done on 42 articles. 13 articles were irrelevant and we excluded them. Lastly, 29 articles were included for systematic review and meta-analysis. A flow diagram depicting this selection process is shown in Figure 1.

### Primary characteristics of the studies

We included 29 eligible articles in the systematic review and meta-analysis for the assessment of the association between OPMDs and the risk of OC. Primary characteristics of all 29 articles included in the systematic review and meta-analysis are thoroughly outlined in Table 1. The eligible articles were published between 2010 and 2023 with a total population of 102,273, of these, 17,501 were patients with OPMDs. Among these 17,501 patients, 15,602 were male and 1,889 were female. Twenty-one (72.4%) studies were cohort studies, three (10.3%) were cross-sectional, three (10.3%) were descriptive, and two (6.8%) were case-control studies. Of the 29 articles, 10 articles were from Europe, eight articles from America, and 11 studies from Asia. A total of six OPMDs were reported: Leukoplakia (26 studies), Erythroplakia (eight studies), OED (seven studies), OLL (three studies), LP (six studies), and OSF (five studies). The mean age of OPMD cases ranged between 18 and 69 years. Most of the etiological factors involved in OPMDs were betel quid chewing, alcohol, and cigarette smoking. Malignant transformation rate as indicative of the development risk of OC was reported in 23 studies. Six studies did not report the MTR. Of the studies, 24 reported the time of OPMD follow up from 6.3 to 540 months. Five studies did not present the time of OPMD follow up. Generally, 89% of the included studies were associated with Leukoplakia. Most OPMDs were identified as oral lesions. In addition, we carried out a quality assessment of the included studies using the NOS scale. Nineteen (66%) studies were of high quality and 10 (34%) studies had a moderate methodological quality. None were of a low quality. The details of the quality assessment are shown in Table 2.

**Table 1:** Primary characteristics of studies included in the systematic review and meta-analysis.

First author's name (Publication year)	Geographical area	No. Case	TP	Sex (M/F)	Age (year)	Study type	Duration of follow up (month)	Type of lesion	Risk factors	Diagnosis	T time to malignant (month)	MTR
Amarasinghe H et al., 2010	UK	403	1029	240/163	>30	CC	NR	OLOSF OLP	Betel-quid chewing Alcohol Smoking	NR	NR	9.33
Brouns E.R.E.A et al., 2013	Netherlands	79	144	44/100	59	RC	51.2	OL	Tobacco Alcohol	Size of the lesion ≥4 cm	57	2.6
Yang YH et al., 2010	Taiwan	340	587	137/200	35–65	RC	6.3	OL OSF	Betel quid chewing Smoking	WHO guideline	NR	NR
Mehrotra D et al., 2012	India	3	15	12/3	45	RC	36–60	PVL	NR	Biopsy	NR	0
Warnakulasuriya S et al., 2011	USA	1,261	1,357	530/827	47	RC	120	OL OLL OLP OED	NR	WHO guideline	108	2.6
Queiroz et al., 2014	Brazil	54	6,560	24/30	56.9	RS	NR	OL E	Smoking Alcohol	Lesion	NR	0.65
Kumar S et al., 2015	India	340	1,241	656/585	>18	CS	NA	OL E OLP	Betel nut chewing Smoking Alcohol	Lesion	NA	NA
Chuang SL et al., 2018	Taiwan	7,663	8,501	7663M	47	PC	67	OL E OSF	Betel nut chewing Smok$ing Alcohol	NA	68.4	8.4
Öhman J et al., 2023	Finland	9	20	9 M	69	RC	33.1	E OED	Smoking	Lesion	73	NR
Liu W et al., 2012	China	57	320	20/37	54.1	RC	61.2	OL	Smoking Alcohol	Lesion	54	17.8
Mendez M et al., 2012	Brazil	137	6,831	70/67	30–49 ≥50	RC	NR	OL	Smoking Alcohol	Lesion	NR	2.46
García-Chías B et al., 2014	Spain	40	116	10 on 30	62.3	RC	44	OL	Tobacco	Lesion	NR	NR
Thennavan A et al., 2015	India	7	7	1 on 6	63.7	RC	24	PVL	Tobacco	Lesion	24	14.29
Starzyńska A et al., 2014	Poland	204	55,911	100/104	58.1	RC	>6	OL	Smoking Alcohol	Dysplasia	>6	0.36
Garcia-Pola MJ et al., 2016	Spain	14	14	3/11	56.4	D	174	OLP	NR	Dysplasia	48	NR
Flores et al., 2016	Brazil	15	30	15 F	68.1	CC	72	OL OED	Tobacco Alcohol	Lesion	NR	NR
Idris AM et al., 2016	Saudi Arabia	26	303	12 on 14	65	D	NA	OED	Shammah user	Lesion	NR	3.64
Ferreira AM et al., 2016	Brazil	410	1,385	648/737	53	D	>540	OPMD	Smoking Alcohol Sunlight	Rural workers	NR	29.6
Fracds et al., 2017	United Kingdom	23	48	12/11	65	C	98.2	OL	Tobacco Alcohol	Lesion	23.4	48
Villa A et al., 2018	USA	30	42	7/35	68.5	RC	24	PVL	Smoking Areca nut Alcohol	Lesion	37	71.43
Upadhyaya J et al., 2018	USA	20	20	6/14	64	RC	91.8	OL	Tobacco	Lesion	78	45
Thomson PJ et al., 2018	China	118	590	99/99	62.3	RC	48	OL OLL	Tobacco Alcohol	Dysplasia Inflammation	48	2.5
Bagan J et al., 2019	Spain	30	63	5 on 25	66.07	RC	72	OL	Tobacco	Lesion	72	63.9
Jayasooriya PR et al., 2020	Sri Lanka	38	93	73/20	>50	C	30	OL OED	NA	WHO guideline	30.6	15.7
McParland H et al., 2020	United Kingdom	51	51	26/25	51	RC	≤120	OL OLL	Smoking Areca nut Alcohol	Dysplasia Keratosis hyperplastic candidosis	120	21.57
Chaturvedi A et al., 2020	USA	1,888	4,886	1,083/805	57.8	RC	55.4	OL	Smoking, Alcohol HIV serostatus	ENT clinician	120	32.2
Abdullah Jaber M et al., 2020	United Arab Emirates	21	395	9/11	52.6	CS	40	OED	Tobacco Alcohol	Biopsy	39.6	5.5
Chiu SF et al., 2021	Taiwan	4,154	11,594	4043/111	49.4	C	33.56	OL E OSF OLP	Betel nut chewing smoking	Dentists and otolaryngologists	21.84	2.68
Gilvetti C et al., 2021	United Kingdom	66	120	60/60	61	RC	62	OL, E	Tobacco Alcohol	Incisional biopsy	50	17.8

TP, total population; NR, non-reported; NA, not applicable; RC, retrospective cohort; RS, retrospective cross-sectional; CC, case control; C, cohort; CS, cross-sectional; OL, oral leukoplakia; E, erythroplakia; PVL, proliferative verrucous leukoplakia; OSF, oral submucous fibrosis; OED, oral epithelial dysplasia; OLP, oral lichen planus; OPMD, oral potentially malignant disorders; OLL, oral lichenoid lesion.

**Table 2:** Methodology quality assessment of included studies using NOS.

Authors	Year	Selection	Comparability	Outcome	Final score	Risk of bias
Amarasinghe H et al.	2010	3	2	3	8	High
Brouns E.R.E.A et al.	2013	4	2	3	9	High
Yang YH et al.	2010	4	2	3	9	High
Mehrotra D et al.	2012	3	2	3	8	High
Warnakulasuriya S et al.	2011	4	1	3	8	High
Queiroz et al.	2014	3	1	3	7	High
Kumar S et al.	2015	4	1	3	8	High
Chuang SL et al.	2018	2	1	3	6	High
Öhman J et al.	2023	2	1	3	6	High
Liu W et al.	2012	2	1	2	5	Moderate
Mendez M et al.	2012	2	1	2	5	Moderate
García-Chías B et al.	2014	3	1	1	5	Moderate
Thennavan et al.	2015	4	1	2	7	High
Starzyńska A et al.	2014	4	2	3	9	High
Garcia-Pola MJ et al.	2016	3	1	3	7	High
Flores et al.	2016	4	1	3	8	High
Idris AM et al.	2016	2	1	2	5	Moderate
Ferreira AM et al.	2016	3	1	1	5	Moderate
Fracds et al.	2017	4	1	3	8	High
Villa A et al.	2018	3	1	1	5	Moderate
Upadhyaya J et al.	2018	3	1	1	5	Moderate
Thomson PJ et al.	2018	4	1	3	8	High
Bagan J et al.	2019	3	1	3	7	Moderate
Jayasooriya PR et al.	2020	3	1	3	7	Moderate
McParland H et al.	2020	4	1	2	7	Moderate
Chaturvedi A et al.	2020	4	1	3	8	High
Abdullah Jaber M et al.	2020	3	2	3	8	High
Chiu SF et al.	2021	3	2	3	8	High
Gilvetti C et al.	2021	3	2	3	8	High

### Meta-analysis

**Figure 2:**
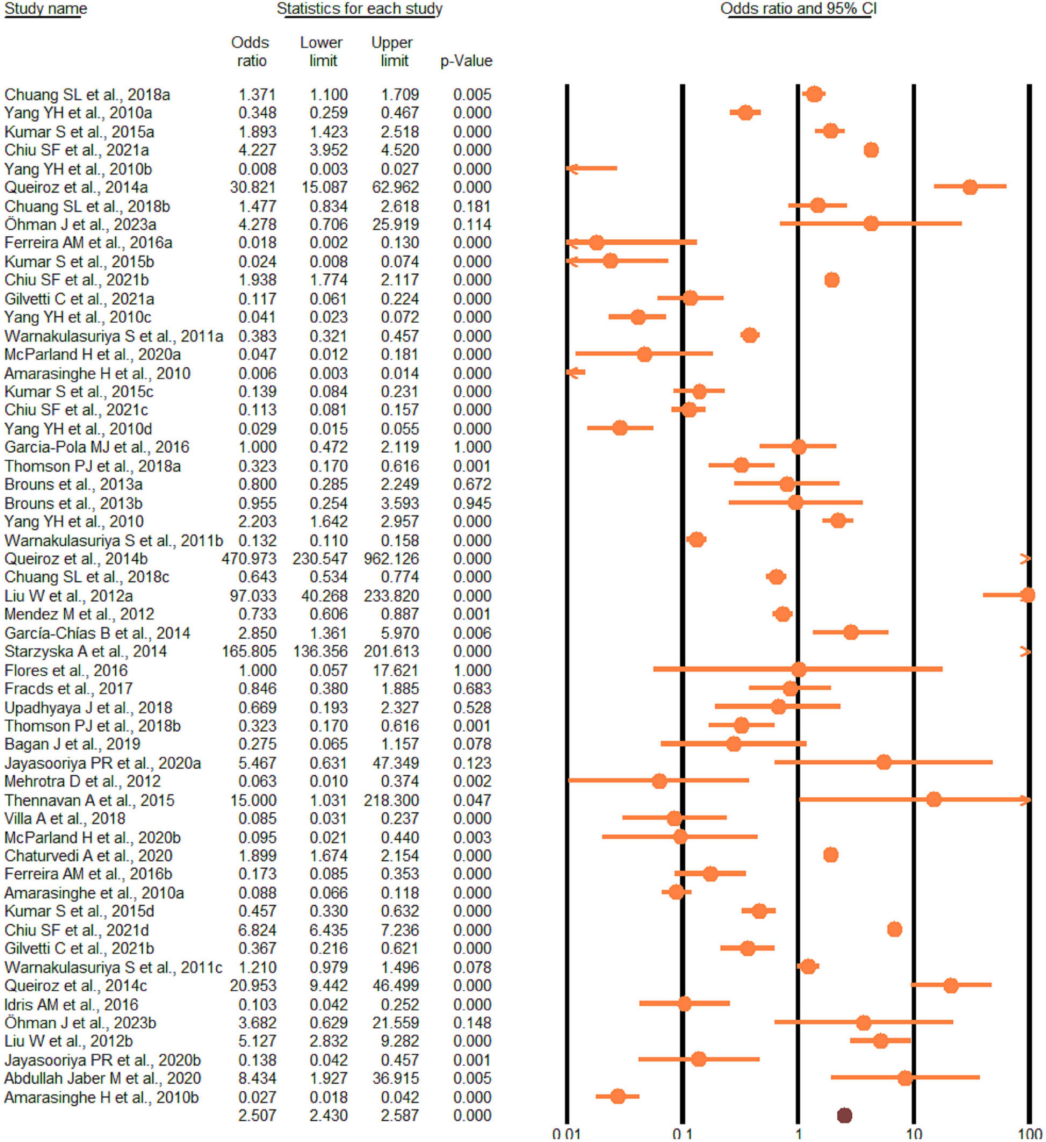
Forest plot of pooled analysis of primary studies reporting the OPMDs and the risk of oral cancer.

**Figure 3:**
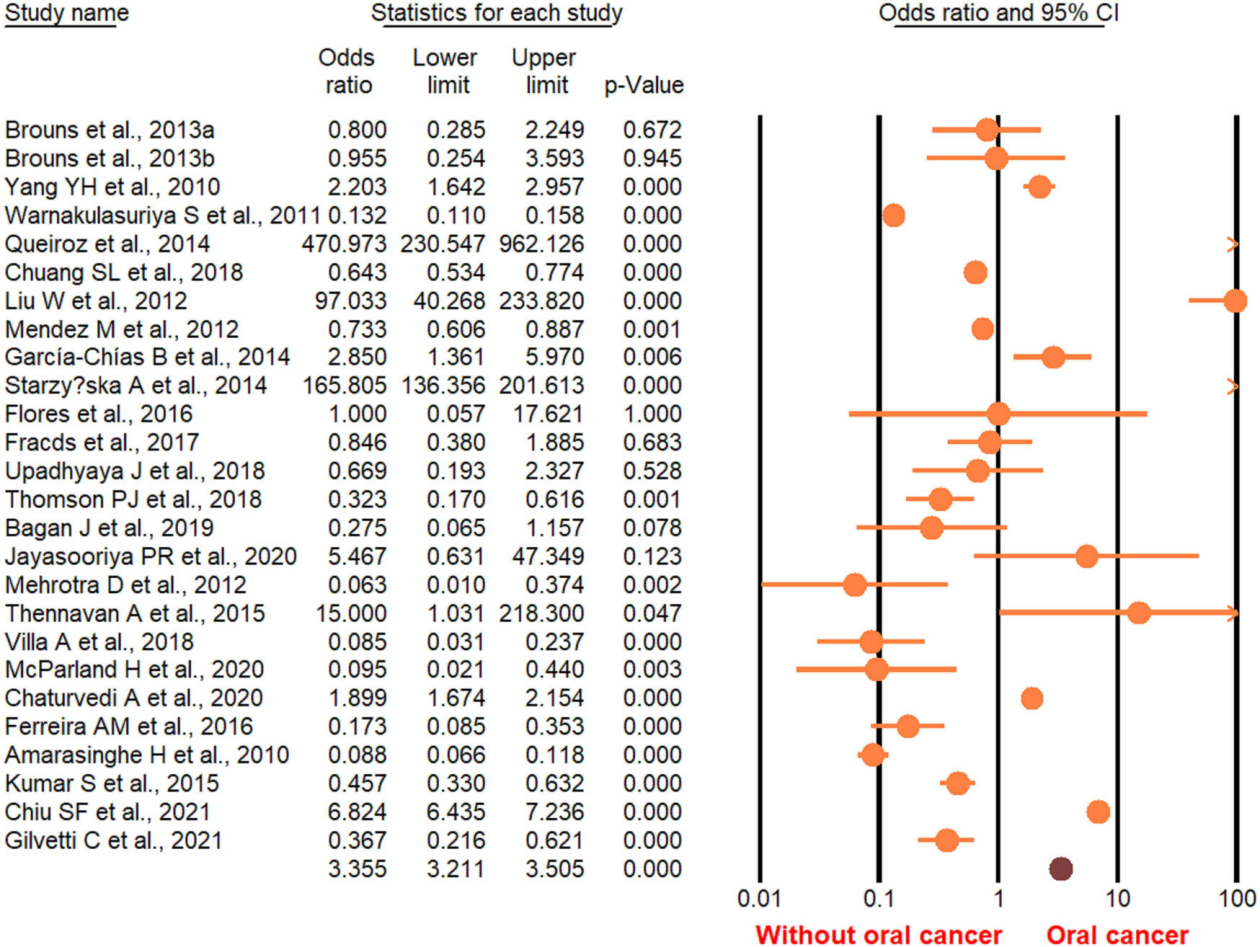
Forest plot of the studies reporting oral leukoplakia and the risk of oral cancer.

**Figure 4:**
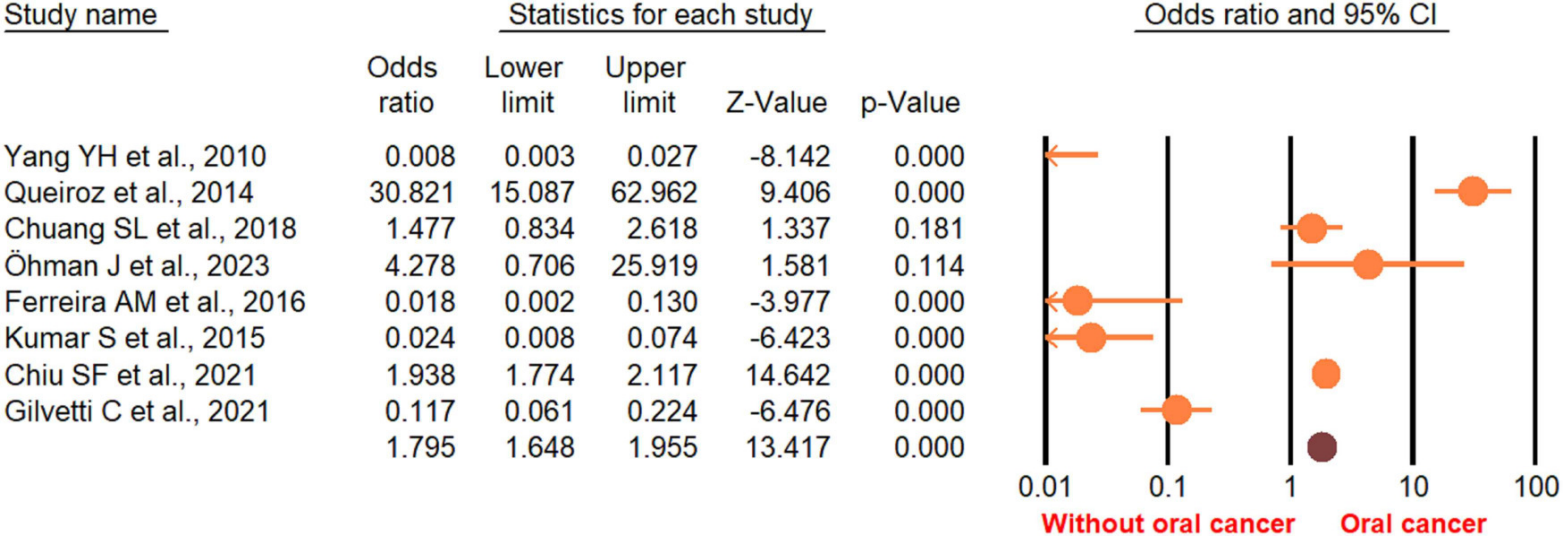
Forest plot of the studies reporting oral erythroplakia and the risk of oral cancer.

**Figure 5:**
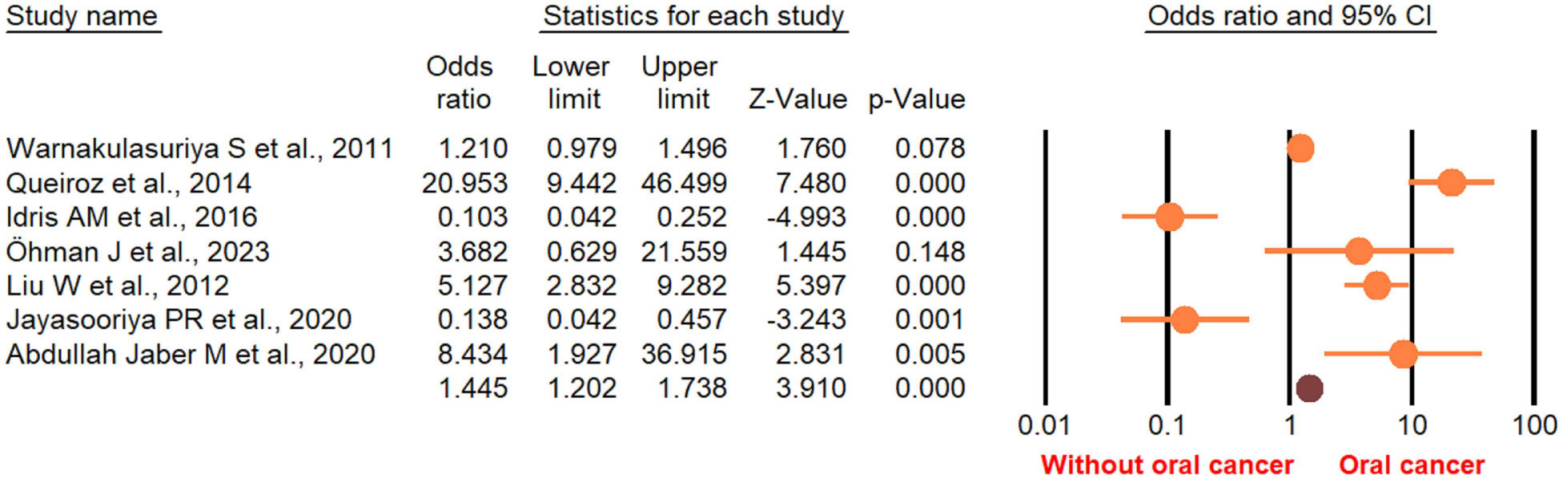
Forest plot of the studies reporting oral epithelial dysplasia and the risk of oral cancer.

**Figure 6:**
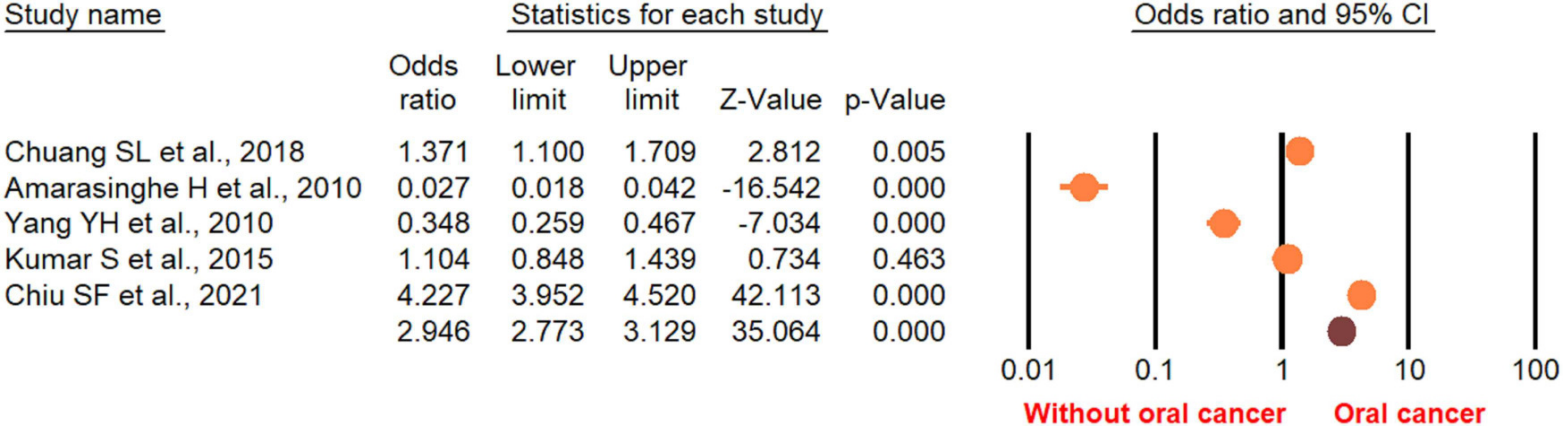
Forest plot of the studies reporting oral submucosa fibrosis and the risk of oral cancer.

**Figure 7:**
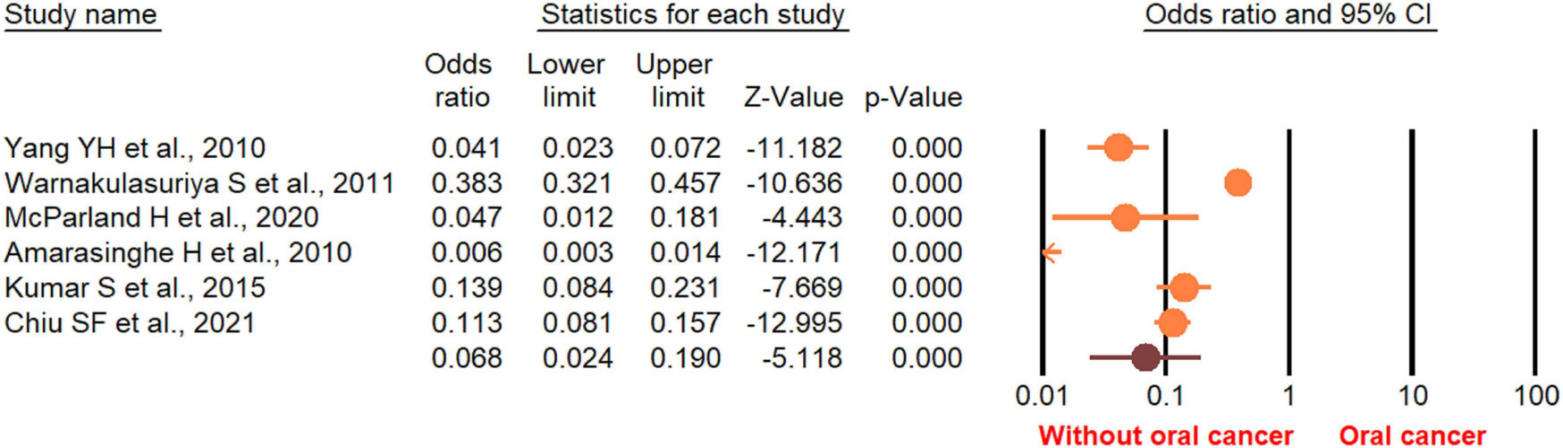
Forest plot of the studies reporting lichen planus and the risk of oral cancer.

**Figure 8:**
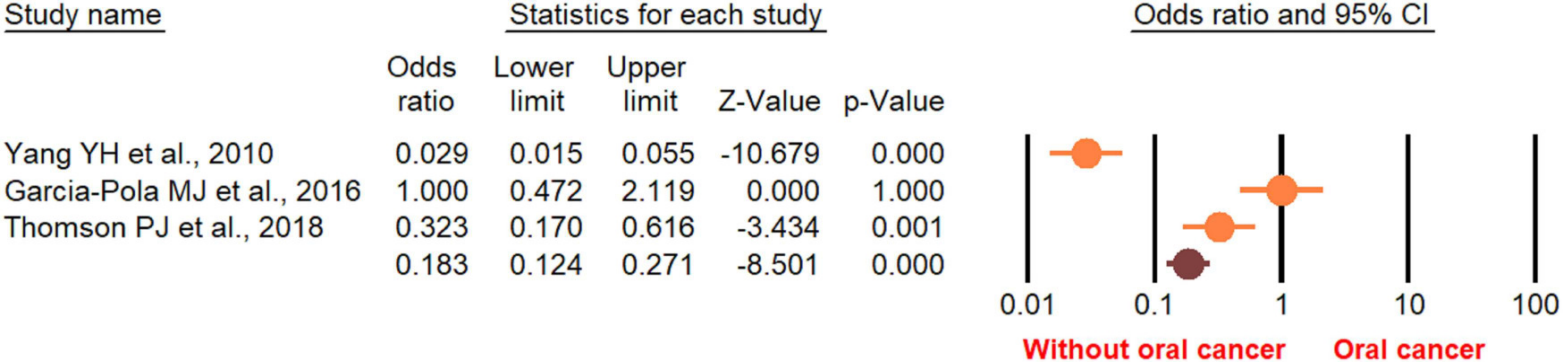
Forest plot of the studies reporting oral lichenoid lesion and the risk of oral cancer.

According to the random-effect model, the pooled analysis showed a significant positive association between OPMDs and the risk of OC [summary OR = 2.50 (95% CI 2.43–2.58), *P* < 0.001] (Figure 2). On the other hand, distinct analysis of patients with oral leukoplakia [summary OR = 3.35 (95% CI 3.21–3.50), *P* < 0.001] (Figure 3), oral erythroplakia [summary OR = 3.35 (95% CI 3.21–3.50), *P* < 0.001] (Figure 4), OED [summary OR = 1.41 (95% CI 1.20–1.73), *P* < 0.001] (Figure 5), and OSF [summary OR = 2.94 (95% CI 2.70–3.12), *P* < 0.001] (Figure 6) were at a higher risk of acquiring OC. Strikingly, we found a decreased risk of OC in patients with OLP [summary OR = 0.068 (95% CI 0.024–0.19), *P* < 0.001] (Figure 7) and OLL [summary OR = 0.18 (95% CI 0.12–0.27), *P* < 0.001] (Figure 8). Significant heterogeneity was detected overall and within all subgroups.

**Figure 9:**
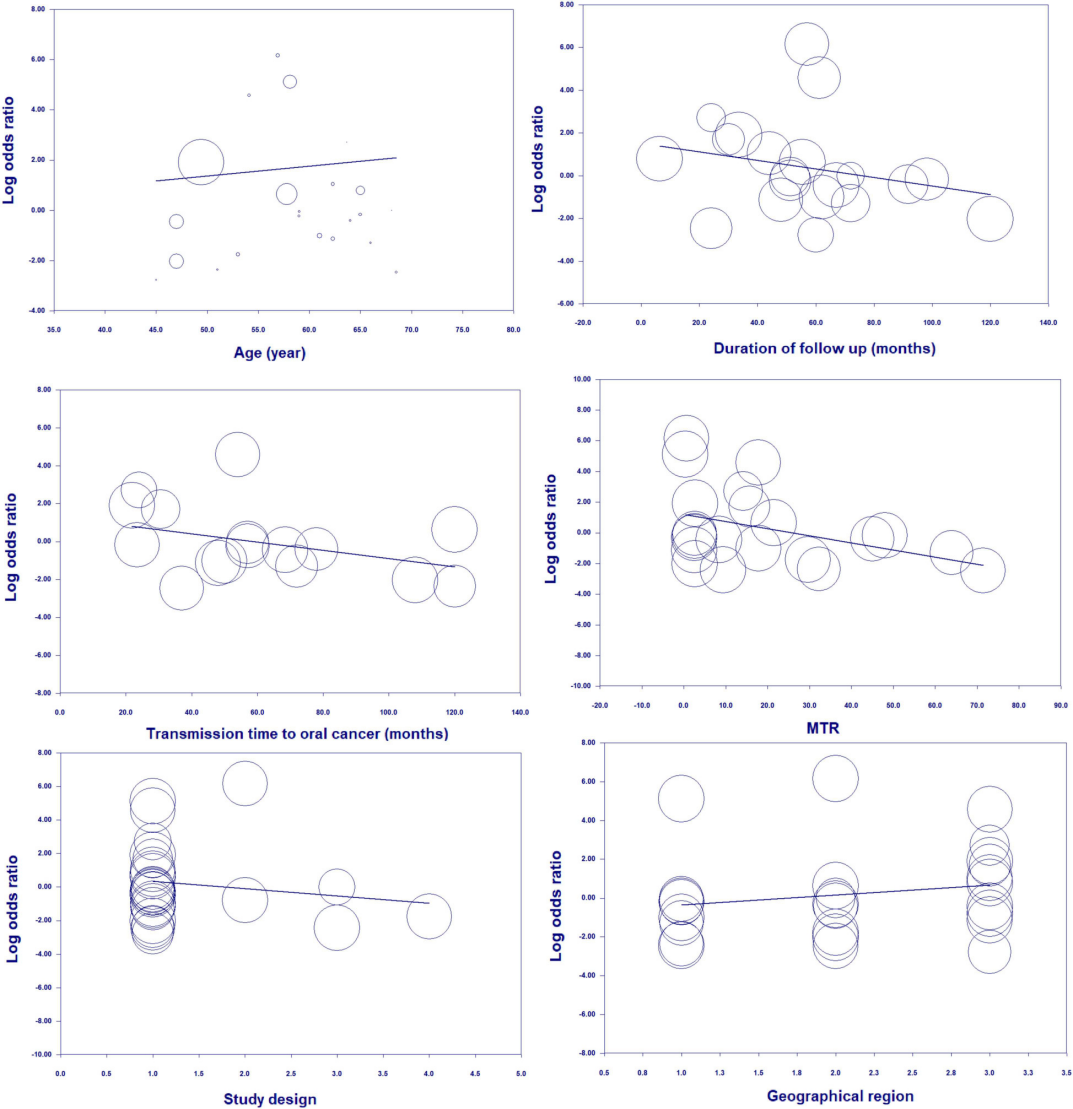
Random-meta-regression of age, duration of follow up, transmission time to cancer, MTR, study design, and geographical region on the risk of oral cancer.

With respect to age, the risk of OC in patients with OPMDs was higher in the population under 60 years. According to the duration of OPMDs follow up and transformation time to cancer, the OC risk was high both in <60 and in >60 months. Moreover, the transformation rate from OPMDs to OC was shorter in MTR <10 percent than MTR >10 percent. The risk of OC in individuals with OPMDs was high in all geographic regions. Based on study design, the OC risk in patients was more statistically significant in a cohort study design relative to other types of study design. Subgroup analysis is presented in detail in Table 3. Random-effects meta-regression showed a significant association between the risk of OC and age, duration of OPMDs follow up, and MTR (Figure 9). A detailed meta-regression is summarized in Table 4.

**Table 3:** Evaluation of the association between OPMDs and the risk of oral cancer using subgroup analysis.

Subgroup	Number of comparisons	OR (95% CI)	*P*-value	Test of heterogeneity		
				*Q*-value	I^2^ (%)	*P*
Overall	29	2.5 (2.43, 2.58)	<0.001	8,363	99.3	<0.001
Age (year)						
<60	15	3.52 (3.3, 3.7)	<0.001	5,034	99.7	<0.001
≥60	14	1.08 (0.88, 1.3)	0.42	92.6	89.2	<0.001
Duration of follow up (month)						
<60	12	6.5 (6.2, 6.8)	<0.001	1,792	99.3	<0.001
≥60	12	0.32 (0.28, 0.36)	<0.001	323.7	96.9	<0.001
NR	5	–	–	–	–	–
Transformation time to oral cancer (month)						
<60	11	8.1 (7.71, 8.6)	<0.001	1,343	99.2	<0.001
>60	8	0.75 (0.68, 0.82)	<0.001	569.2	99.1	<0.001
NR	10	–	–	–	–	–
MTR (%)						
<10	11	4.05 (3.8, 4.2)	<0.001	4,646	99.8<	<0.001
>10	11	0.92 (0.33, 2.5)	0.88	217	95.3	<0.001
NR	7	–	–	–	–	–
Study design						
Cohort	21	3.7 (3.6, 3.9)	<0.001	4,217	96.2	<0.001
Case control	2	0.09 (0.06, 0.12)	0.14	2.71	63.2	0.09
Cross-sectional	3	14.5 (0.01, 13,060)	0.44	300	99.5	<0.001
Descriptive	3	1.43 (0.05, 1.48)	0.38	562	98.3	<0.001
Geographical region						
Europe	10	8.7 (7.5, 10.1)	<0.001	2,097	99.6	<0.001
America	8	0.83 (0.76, 0.90)	<0.001	898	99.2	<0.001
Asia	11	4.9 (4.65, 5.18)	<0.001	952.4	99.0	<0.001

OR, odds ratio; MTR, malignant transformation rate.

**Table 4:** Random meta-regression analysis for covariates involved in OPMD and risk of oral cancer.

Moderators	β-coefficient	SE	*Z*-value	95% CI	2-sided *P*-value
Age (year)	0.039	0.005	7.65	0.02–0.04	<0.001
Duration of follow up (months)	−0.020	0.01	−1.98	−0.03 to −0.0002	0.047
Transformation time to oral cancer (month)	−0.021	0.014	−1.50	−0.05–0.006	0.13
Malignant transformation rate (%)	−0.046	0.023	−2.00	−0.091 to −0.0008	0.045
Study design	−0.43	0.50	−0.87	−1.42–0.54	0.38
Geographical region	0.50	0.55	0.90	−0.58–1.59	0.36

SE, standard error; OPMD, oral potentially malignant disorders; CI, confidence interval.

Sensitivity analyses were conducted by sequentially excluding each study, beginning with the one that had the largest sample size and progressing to the smallest, utilizing the “leave-one-out” approach. Interestingly, when the studies with the largest sample size to smallest sample size were removed, the same results were obtained, suggesting strong consistency of the total effect size in this meta-analysis.

**Figure 10:**
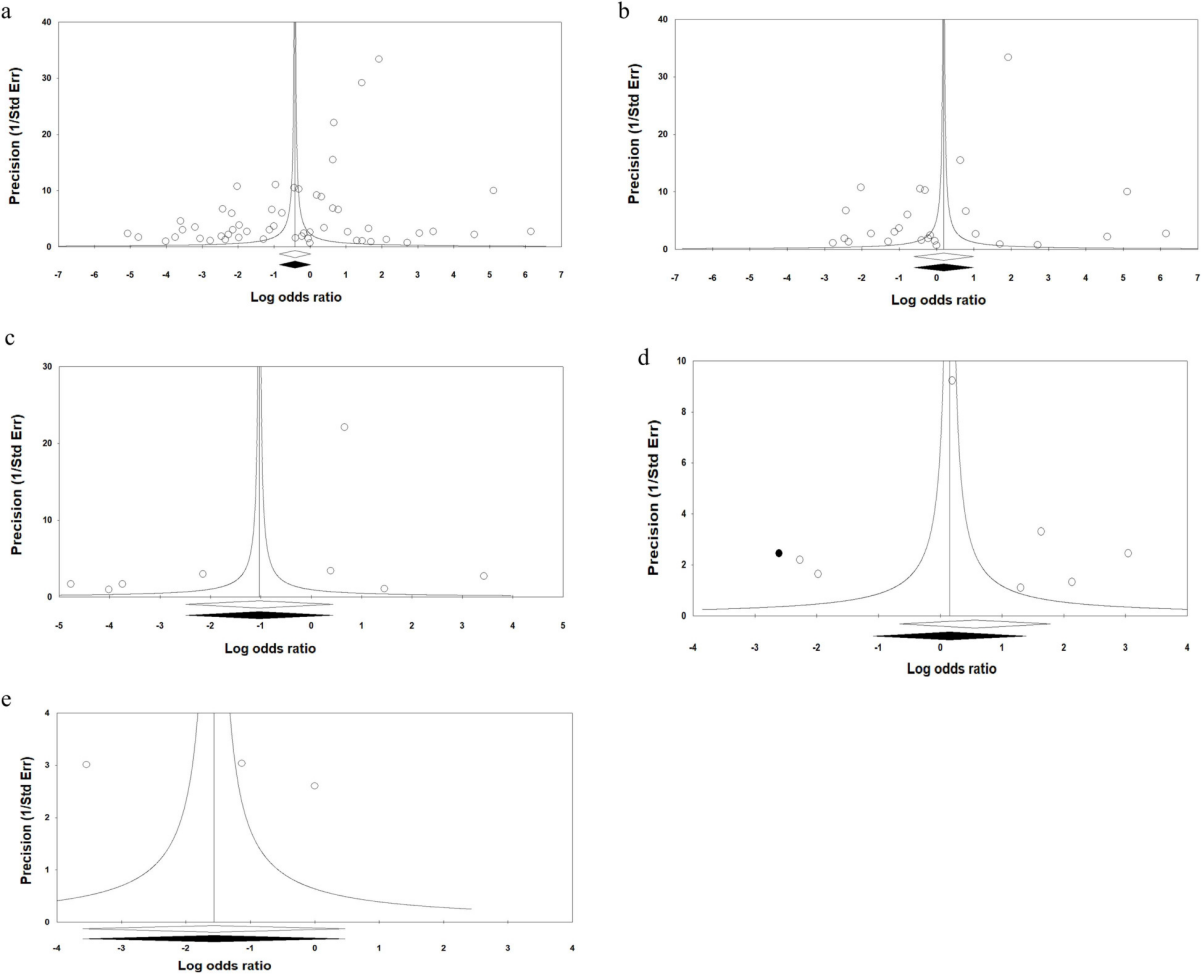
Funnel plots assessing the publication bias in studies included in the meta-analysis, **(a)** overall analysis of OPMDs, **(b)** oral leukoplakia, **(c)** oral erythroplakia, **(d)** OED, and **(e)** OSF.

The funnel plot suggests publication bias for the overall results of OPMDs using the Begg's rank correlation (Kendall s Tau with continuity correction = 0.22; Z = 2.47; 2-tailed *P*-value = 0.013) and Egger's linear regression (intercept = −6.6; standard erro*r* = 1.99; 95% CI = −10.6, - 2.6; *t*-value = 3.32; df = 28; 2-tailed *P*-value = 0.001) (Figure 10a). There was no publication bias for Leukoplakia (Figure 10b), Erythroplakia (Figure 10c), OED (Figure 10d), or OLL (Figure 10e). These findings were confirmed by the Egger test. The trim-and-fill method was employed to detect and adjust imputed missing studies for publication bias in asymmetry funnel plot. The imputed pooled effect size was similar to the overall effect size. The “classic fail-safe N” approach indicated that 657 theoretically absent studies are required to render the estimated effect size statistically non-significant.

## Discussion

To our knowledge, this is the first systematic review and meta-analysis that covers the association between all premalignant oral lesions with a high incidence of OC. Therefore, this systematic review and meta-analysis aimed to estimate the risk of acquiring OC caused by OPMDs such as Leukoplakia, Erythroplakia, oral epithelial dysplasia, OLL, and OLP. We included 29 studies that evaluated the risk of OC in patients with OPMDs. The results of this study show a statistically significant increased risk of OC among individuals with Leukoplakia, Erythroplakia, OED, and OSF. The OC risk was reduced, however, in individuals with LP and OLL. In addition, a duration less than 60 months with MTR <10% showed the highest incidence of developing OC. Taken together, the transformation of OPMDs to OC could be more likely in patients under the age of 60 with a long-time usage of risk factors such as smoking, alcohol consumption, and betel-quid chewing. Multiple studies have shown that the development of increased risk of OC is strongly associated with OPMDs such as Leukoplakia, Erythroplakia, and OED (25, 37–39).

Oral potentially malignant disorders (OPMDs) is the term applied by the WHO for precancer or premalignant lesions that are prone to transformation to OC (40, 41). OPMDs are associated with betel-quid chewing, alcohol consumption, and smoking (42, 43). As, the ethnic groups of Asian-pacific region, smokeless tobacco and areca nut composed of a vast majority of the most common OPMD (44). Oral leukoplakia, erythroplakia, submucous fibrosis, oral epithelial dysplasia, and lichen planus are part of the spectrum of OPMD (45). However, quitting smoking and betel nut chewing could decrease 36% and 62% of cases of OPMDs, respectively, and 26% of malignant transformations to OC could be prevented if betel quid was no longer consumed (46). One Two the major OPMDs are leukoplakia and erythroplakia. and they are considered significant potentially malignant lesions. Leukoplakia is characterized by a white patch or plaque that cannot be removed through rubbing and that does not fit the clinical or histopathological criteria for any other disease. Moreover, it is also the most common and extensively researched type (47, 48). Erythroplakia is defined as a red macule or plaque that is not the same clinically and histopathologically to any other lesion. Although it occurs less often, it may exhibit high potential for malignant transformation to OC (49). Several factors have been presented to predict an elevated risk of oral cancer such as age, sex, tobacco habits, homogeneity and lesion size, site of oral disorder, and severity of epithelia dysplasia (50, 51). Another important OPMD is oral epithelial dysplasia (OED). OED is characterized by a spectrum of epithelia damages caused by an accumulation of genetic changes and is associated with an elevated risk of transformation to OSCC (52, 53). OED can produce a wide range of chromatic and textural changes and cause lesions such as leukoplakia, erythroplakia, and oral submucous fibrosis (54, 55). To prevent the development of SCC, OED requires surgical excision (56). However, developed OED in the oral mucosa could be indictive of a progression to invasive oral cancer (57).

There was a substantial heterogeneity in studies included in this meta-analysis. Therefore, to explore the source of heterogeneity between selected studies, subgroup analyses were performed. Firstly, we examined the incidence of oral cancer in subgroups of age < and >60 years in patients with premalignant oral lesions. The results indicated the highest incidence of developing oral cancer was in age <60 years. In addition, to account for the effect of MTR, a known covariate within this context, we determined the incidence of OC in two subgroups, including one which involved studies that had MTR <10% and another which involved studies that had MTR >10%. Subgroup analysis of MTR <10% revealed the highest incidence of developing OC in patients with OPMDs. On the other hand, we found an elevated risk of OC in subgroups of studies with a cohort study design and in all geographic regions.

In the next step we indicated that covariates of age and MTR have a significant impact on this association, and this result was also confirmed by meta-regression analysis. Strikingly, meta-regression analysis indicated a strong association between the incidence of developing oral cancer in age and MTR. In the present study, our results confirm that age and MTR are potential confounding covariates that can affect the risk of OC in patients with OPMDs. Our findings show that age, duration of follow up, and MTR have a considerable impact on risk.

One notable strength of this study is that it was a systematic search through electronic databases, and it contained a high number of studies with a precise protocol. Moreover, this systematic review was performed based on the standard methodology of GRADE.

## Limitations

This study has several limitations. Firstly, our study is influenced by the biases that were prevalent in the original studies that were included. Second, significant heterogeneity was observed in our meta-analyses due to the cohort study design, duration of follow up, MTR less than 10 percent, transmission time to cancer, and the variables such as age and geographical region that varied widely among the different studies. Third, data on the pooled OR with their 95% CIs of variables associated with OPMDs were not presented in certain studies. To acquire information concerning the missing data, we contacted the relevant authors of the original studies; however, none of the authors responded. A differing combination of risk factor influences from studies with different OPMDs is another limitation in this study. Therefore, given the limitations, the findings of the present meta-analysis should be interpreted with caution.

## Conclusion

In the present study, we found that the highest incidence rates of developing oral cancer came from the premalignant oral lesions OL, OE, OED, and OSF. Age and MTR are the most important factors in developing oral carcinoma followed by the premalignant oral lesion. However, OPMDs are fairly prevalent conditions encountered by general practitioners and head and neck and oral medicine specialists in their daily practice. It is crucial to enhance public health awareness regarding the risks of age and MT rates and to encourage monitoring of these lesions; concurrently, effective communication with the patient is of paramount significance. Following this guidance would help in reducing the transformation of these oral conditions into invasive cancer.

## Data Availability

The original contributions presented in the study are included in the article/Supplementary Material, further inquiries can be directed to the corresponding author.
