# Supplementary figures and images for: Malignant transformation rate of oral precancerous disorders to oral cancer: systematic review and meta-analysis of the current evidence
Source: Front Oral Health. 2025 Oct 2;6:1673474. doi: 10.3389/froh.2025.1673474 (PMC12528030; doi:10.3389/froh.2025.1673474)

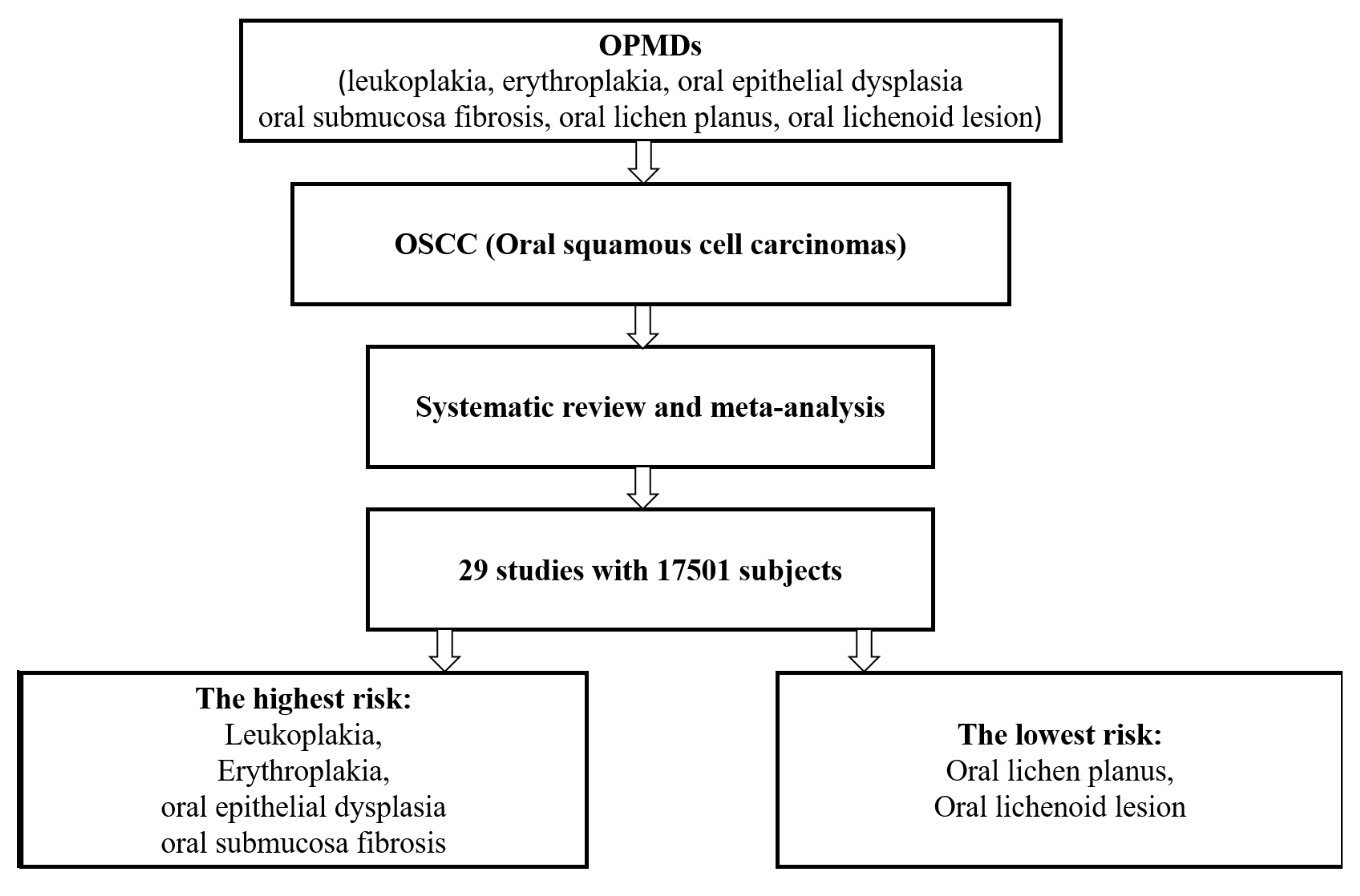

Supplement: Supplementary file 1 [file Image1.tif]
