# Supplementary material for: Malignant transformation rate of oral precancerous disorders to oral cancer: systematic review and meta-analysis of the current evidence
Source: Front Oral Health. 2025 Oct 2;6:1673474. doi: 10.3389/froh.2025.1673474 (PMC12528030; doi:10.3389/froh.2025.1673474)
Supplement: Supplementary file 3 [file Table2.docx]

**Malignant transformation rate of oral precancerous disorders to oral cancer: Systematic review and meta-analysis of the current evidence**

**Maryam Khodadadi^1*^, Mohammadmehdi Khodadadi^2^**

^1^Xi'an Jiaotong University, School of Stomatology, Xi'an, Shaanxi Province, China.

^2^Medical Campus, Zhengzhou University, Zhengzhou, Henan Province, China.

***Corresponding author:**

Maryam Khodadadi, Xi'an Jiaotong University, School of Stomatology, Xi'an, Shaanxi Province, China. E-mail address: [maryamkhodadadi539@gmail.com](mailto:maryamkhodadadi539@gmail.com),

[maryam.khodadadi5425@gmail.com](mailto:maryam.khodadadi5425@gmail.com).

**Box.1**

1. **Database Search Strategies**

**PubMed**

((((((((((((Oral potentially malignant disorders[Title/Abstract]) OR ((((((OPMDs[Title/Abstract]) OR leukoplakia [Title/Abstract]) OR oral leukoplakia [Title/Abstract]) OR Proliferative verrucous leukoplakia [Title/Abstract]) OR oral erythroplakia [Title/Abstract]) OR erythroplakia [Title/Abstract]))) OR oral epithelial dysplasia) OR OED [MeSH Terms])) OR (((OPMD [MeSH Terms]) OR oral submucosa fibrosis [Title/Abstract]) OR OSF[Title/Abstract]))) OR ((((((((((((cohort studies[MeSH Terms]) OR cross-sectional studies[MeSH Terms]) OR case-control studies[MeSH Terms]) OR randomized controlled trial[Publication Type])) OR (((((oral planus[Title/Abstract]) OR ((LP[Title/Abstract]) OR oral lichenoid lesion [Title/Abstract])) OR ((OLL [Title/Abstract]) OR oral malignant cancer [Title/Abstract])) OR ((Asia[Title/Abstract]) OR Europe [Title/Abstract])) OR ((America [Title/Abstract]) AND lesion type [Title/Abstract]))) OR (((clinical trial[Publication Type]) OR "clinical trials as topic"[MeSH Terms]) OR "double-blind method"[MeSH Terms])) OR ((((randomized[Title/Abstract]) OR randomized [Title/Abstract])) OR ((trial[Title/Abstract]) OR trials[Title/Abstract]))) OR ((dysplasia [Title/Abstract]) OR ((((randomized[Title/Abstract]) OR randomized [Title/Abstract])) OR ((trial[Title/Abstract]) OR trials[Title/Abstract])))) OR ((((blind[Title/Abstract]) OR mask*[Title/Abstract])) OR ((((((single[Title/Abstract]) OR double[Title/Abstract]) OR doubled[Title/Abstract]) OR triple[Title/Abstract]) OR tripled[Title/Abstract]) OR treble[Title/Abstract])))) OR ((systematic review[Title/Abstract]) OR meta-analysis[Title/Abstract])))) OR English [Language])) NOT "Case Reports"[Publication Type]

**Embase**

('oral potentially malignant disorders/exp OR 'oral malignant disorders':ab,ti OR 'leukoplakia':ab,ti OR 'oral leukoplakia':ab,ti OR 'erythroplakia':ab,ti OR 'oral erythroplakia':ab,ti OR 'opmd':ab,ti OR 'oed':ab,ti) OR ('lp':ab,ti OR (oll OR cancer:ab,ti) OR 'oral epithelial malignancy':ab,ti OR 'lichen planus':ab,ti OR 'oral lichenoid lesion':ab,ti) OR [humans]/lim OR [english]/lim OR [embase]/lim NOT ([embase]/lim AND [medline]/lim) NOT 'case report'/exp

**Web of Science**

TS = (Oral lesions OR oral potentially malignant disorders OR OPMDs OR leukoplakia OR oral epithelial dysplasia OR oral submucosa fibrosis OR OC OR oral erythroplakia OR oral leukoplakia) OR TS= (OL OR OE* OR OED*)

**Scopus**

(Oral malignant lesions OR OPMDs OR oral potentially malignant disorders OR oral leukoplakia OR oral erythroplakia OR oral epithelial dysplasia OR oral dysplasia OR oral submucosa fibrosis) AND (oral cancer OR oral malignancy lesions OR cancer*).
